# Association of patatin-like phospholipase domain-containing protein 3 gene polymorphisms with susceptibility of nonalcoholic fatty liver disease in a Han Chinese population

**DOI:** 10.1097/MD.0000000000004569

**Published:** 2016-08-19

**Authors:** Guohe Song, Chao Xiao, Kai Wang, Yupeng Wang, Jian Chen, Yang Yu, Zhaowen Wang, Guilong Deng, Xing Sun, Lin Zhong, Chongzhi Zhou, Xiaosheng Qi, Shuyun Wang, Zhihai Peng, Xiaoliang Wang

**Affiliations:** a Department of General Surgery, Shanghai First People's Hospital, School of Medicine, Shanghai Jiao Tong University, Shanghai; b Department of General Surgery, Children's Hospital of Zhengzhou, Henan, P. R. China.

**Keywords:** gene polymorphisms, nonalcoholic fatty liver disease, phospholipase domain-containing protein 3

## Abstract

Gene polymorphisms had been found to be associated with increased risk of nonalcoholic fatty liver disease (NAFLD). The aim of the present study was to assess the association between rs2896019 and rs3810622 in PNPLA3 with the susceptibility to NAFLD in Han Chinese population.

A total of 384 NAFLD patients and 384 controls were enrolled in the study. Blood samples collected from each subject were used for biochemical index analysis and DNA extraction. Genotyping analyses of PNPLA3 rs2896019 and rs3810622 were performed by real-time PCR methods.

Results showed that patients with genotype GG of rs2896019 had a higher incidence of NAFLD than patients with genotypes GT and TT (62.4% vs 52.0% and 43.3%, respectively, *P* = 0.002), and a higher risk of moderate to severe NAFLD than patients with genotypes GT and TT (60.3% vs 46.2% and 40.2%, respectively, *P* = 0.03). Furthermore, patients with genotype GG of rs2896019 had higher levels of low-density lipoprotein (LDL, *P* < 0.001), ALT (*P* = 0.003), and AST (*P* = 0.002). Patients with genotype TT of rs3810622 had a higher incidence of NAFLD than patients with genotypes CT and CC (56.7% vs 48.4% and 41.5%, respectively, *P* = 0.013). Likewise, patients with genotype TT of rs3810622 had higher levels of ALT (*P* = 0.021) and blood glucose (GLU) (*P* = 0.034). Haplotype association analysis showed that GT haplotype conferred a statistically significant increased risk for NAFLD (OR = 1.49; 95% CI = 1.20–1.84, *P* < 0.01).

These results suggest that PNPLA3 rs2896019 and rs3810622 polymorphisms significantly contribute to increased NAFLD risk in Han Chinese population.

## Abstract 2

Supplemental Digital Content is available in the text

## 1 Introduction

Nonalcoholic fatty liver disease (NAFLD) is the leading cause of chronic liver disease worldwide which is characterized by the association of hepatic steatosis with abnormal fat accumulation in liver cells that could progress to cirrhosis.^[1]^ The prevalence of NAFLD is obviously increasing, affecting nearly 20% to 34% of the population in western countries and 12% to 24% in Asian countries.^[2,3]^ NAFLD encompasses a spectrum of pathological processes ranging from simple fatty liver, nonalcoholic steatohepatitis (NASH), fibrosis, and cirrhosis.^[4]^ Although it is well-recognized that obesity, insulin resistance, and metabolic syndrome may influence the development and progression of NAFLD, inherited factors, and in particular single nucleotide polymorphisms (SNPs) in genes could play a significant role in the severity of liver disease and the susceptibility to NAFLD.^[5–7]^

Recent advances in genome-wide association studies (GWAS) have revealed that multiple gene variants might contribute to the pathogenesis of NAFLD. Among these candidate genes, patatin-like phospholipase domain-containing protein 3 (PNPLA3) and several other genes display high association with NAFLD in different ethnic populations.^[5,8,9]^ PNPLA3, belongs to the patatin-like phospholipase family, is a 481 amino acid transmembrane protein which exhibits hydrolase activity against triglycerides.^[10]^ Numerous studies have shown that PNPLA3 may be the most important gene for the occurrence for NAFLD, in particular, a nonsynonymous sequence variation (rs738409 C > G, I148M) in PNPLA3 has been identified to be strongly associated not only with steatosis but also with clinically relevant factors and the progression of NAFLD.^[11,12]^ A GWAS from Kawaguchi et al^[8]^ had revealed several SNPs, including rs2896019 and rs3810622 in the *PNPLA3* gene, were associated with the histological classifications of NAFLD in the Japanese population. Kitamoto et al^[13]^ also discovered that these 2 SNPs were associated with the progression of simple steatosis to NASH and the development of NAFLD. Moreover, a recent study suggested that rs2896019 loci might affect hepatic lipid accumulation.^[14]^

In a previous study, we had demonstrated that rs738409 loci in *PNPLA3* gene was significantly associated with NAFLD (*P* = 0.0002).^[15]^ As no study regarding the association of rs2896019 and rs3810622 and predisposition to NAFLD in other populations has yet been published, in this study, we will further evaluate the association of rs2896019 and rs3810622 polymorphisms in *PNPLA3* gene with NAFLD based on a Han Chinese population, which represents the world's largest population, to further elucidate the genetic causes of NAFLD.

## 2 Materials and methods

### 2.1 Study groups

This study consist of 384 cases (male: n = 229; female: n = 155; and age 45.5 ± 13.1 years), and 384 healthy controls (male: n = 226; female: n = 158; and age 45.3 ± 13.4 years) recruited at the departments of gastroenterology and medical center of Shanghai First People's Hospital from June 2011 to December 2011. All enrolled subjects were unrelated and ethnically Han Chineses. The diagnosis of NAFLD was established based on ultrasonography examinations according to the guideline defined by the Chinese National Consensus Workshop on NAFLD.^[16]^ Ultrasonic diagnosis criteria were: diffuse enhancement of near-field echo in the hepatic region (stronger than in the kidney and spleen region) and gradual attenuation of the far-field echo; unclear display of the intrahepatic lacuna structure; presentation of mild to moderate hepatomegaly with a round and blunt border; color Doppler ultrasonography shows reduced blood flow in the liver, which may be difficult to detect, but the distribution of blood flow is normal; and unclear or nonintact display of the envelope of the right liver lobe and diaphragm. Patients with a mild degree of fatty liver disease illustrate item 1 and any 1 of items 2 to 4; patients with moderate fatty liver disease illustrate item 1 and any 2 of items 2 to 4; and patients with severe degree of fatty liver disease illustrate items 1 and 5 and any 2 of items 2 to 4. Cases with advanced cirrhosis, other causes of liver disease (hepatitis B, hepatitis C), or autoimmune liver disease (AILD) were excluded in this study. Patients with significant alcohol consumption (>140 g/week in men or >70 g/week in women) were also excluded. Approval was obtained by the ethical committees of the Shanghai Jiao Tong University and strictly conforms to the principles of the Declaration of Helsinki.

### 2.2 Specimen collection and genomic DNA isolation

Venous blood samples (5 mL) were collected from patients and healthy participants in the morning. Two milliliter of the blood specimen was stored at −80 °C for DNA extraction, and the remaining was centrifuged for the measurement of the biochemical indicators (total cholesterol [TC], triglyceride [TG], high-density lipoprotein, low-density lipoprotein [LDL], alanine aminotransferase [ALT], aspartate transaminase [AST], total bilirubin, direct bilirubin, gamma-glutamyltransferase [γ-GT], alkaline phosphatase, blood glucose [GLU], and glycated albumin). Genomic DNA was extracted by standard methods using a ClotBlood DNA Kit (Beijing Cowin Biotech Co., LTD., item No.: CW0545), and the DNA concentration was determined by NanoDrop^TM^ 1000 Spectrophotometer (Thermo Fisher Scientific, Copenhagen, Denmark).

### 2.3 Genotyping and real-time PCR

The genotypes of samples were detected by TaqMan SNP genotyping method. The information of the international serial numbers, gene names, location, and sequence of the detection probes are presented in Table S1. Amplification was performed by ABI ViiA™ 7 real-time fluorescent quantitative PCR amplifier (Applied Biosystems, ABI), reaction mixture containing 2.5 μL of 2 × Taqman Genotyping Master Mix, 5 pmol of 40 × TaqMan SNP Genotyping Assay and 20 ng of genomic DNA. Real-time PCR was performed under the following conditions: an initial denaturation step at 95 °C for 10 minutes, followed by 55 PCR cycles of denaturation at 92 °C for 15 seconds, annealing for 1 minutes at 60 °C. The final extension was at 72 °C for 5 minutes.

### 2.4 Statistical analysis

Continuous variables were expressed as mean ± standard deviation (SD) for normally distributed variables. Comparison of categorical variables were presented as counts (frequencies) and tested by Chi-square test. Quantitative data showed equal variance based on population or did not demonstrate normal distribution was analyzed by the Mann–Whitney *U* test or Kruskal–Wallis H test. Odds ratios (ORs) were calculated with 95% confidence intervals (95% CIs) for evaluation of the relative risk of NAFLD. Hardy–Weinberg equilibrium was assessed in the control group by using the Chi-square test. Haplotypes of the 2 SNPs were analyzed by SHEsis software (http://analysis.bio-x.cn). Statistical significance was defined as *P* < 0.05. The SPSS software version 19.0 (SPSS Inc., Chicago, IL) was used for all of the statistical analyses.

## 3 Results

### 3.1 Characteristics of the study population in baseline clinical information

The clinical and biochemical parameters of NAFLD patients and controls are shown in Table 1. There were no significant differences (*P* > 0.05) between the NAFLD patients and normal controls with respect to gender, age, BMI, blood pressure, TC, high-density lipoprotein cholesterol, total bilirubin, direct bilirubin, γ-GT, GLU, or glycated albumin. Compared to controls, NAFLD patients had higher levels of TG and LDL cholesterol, as well as higher occurrence of the risk factors of liver damage, including ALT, AST, and alkaline phosphatase.

**Table 1:**
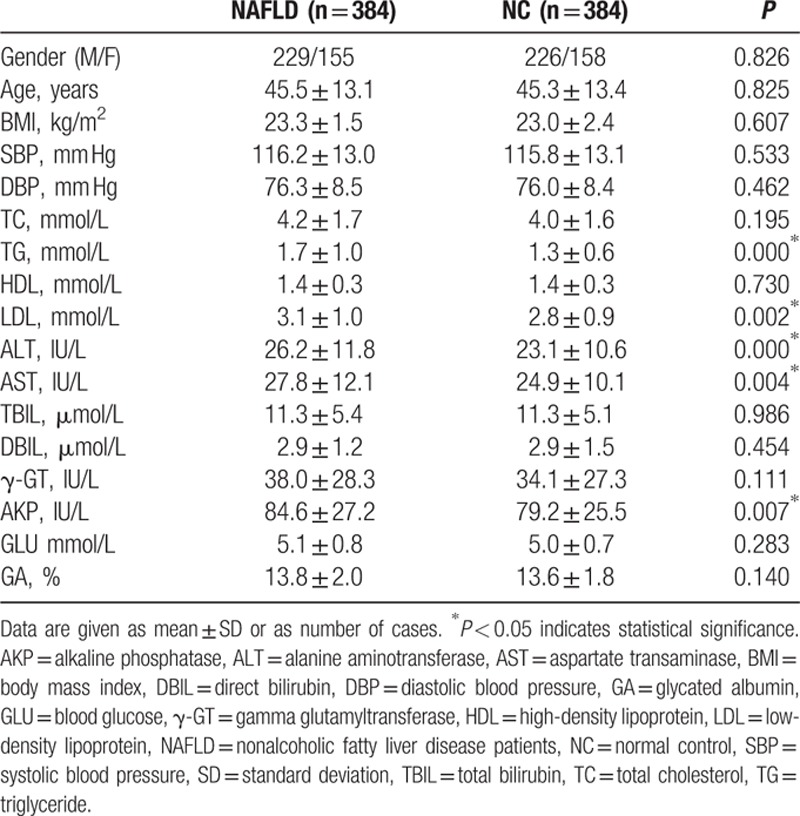
Main characteristics of patients with NAFLD group and controls in baseline information.

### 3.2 Genotype distributions of rs2896019 and rs3810622 in *PNPLA3* gene among the cases and controls

**Figure 1:**
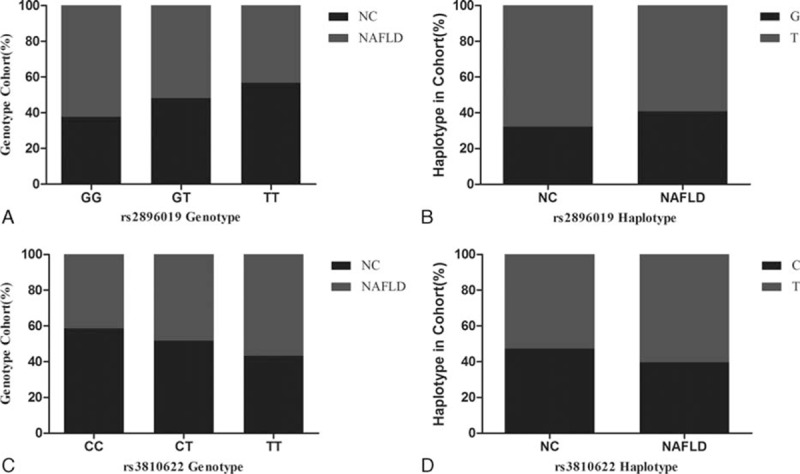
Correlation between controls and nonalcoholic fatty liver disease patients (NAFLD) group in genotype frequency and frequency of allelic gene at loci rs2896019 and rs3810622 of SNP. (A) Patients with genotype GG had higher incidence of NAFLD than patients with genotype GT and TT (62.4% vs 52.0% and 43.3%, respectively, *P* = 0.002). (B) The frequency of allele G was higher in NAFLD group than in the controls (40.8% vs 32.2%, *P* < 0.001). (C) Patients with genotype TT had higher incidence of NAFLD than patients with genotype CT and CC (56.7% vs 48.4% and 41.5%, respectively, *P* = 0.013). (D) The frequency of allele T was higher in NAFLD group than in the controls (60.2% vs 39.8%, *P* = 0.015).

Of the 384 pairs of patients, several patients were excluded in the further analysis for the failure of DNA extraction or SNP genotyping detection. The genotype and allele frequencies of rs2896019 and rs3810622 polymorphism in NAFLD group and controls are summarized in Table 2. Allelic and genotypic frequencies fit with Hardy–Weinberg equilibrium between the study groups. The GG, GT, and TT genotype frequencies of the rs2896019 locus were 16.7% (63), 48.3% (182), and 35.0% (132) among the NAFLD group and 10.0% (38), 44.3% (168), and 45.7% (173) among the controls. Individuals with genotype GG of the rs2896019 had a higher incidence in NAFLD individuals than in controls (62.4% vs 52.0% and 43.3%, respectively, *P* = 0.002, Fig. 1A). The prevalence of allele G was significantly higher in the NAFLD group than in the controls (40.8% vs 32.2%, *P* < 0.001, Fig. 1B).

**Table 2:**
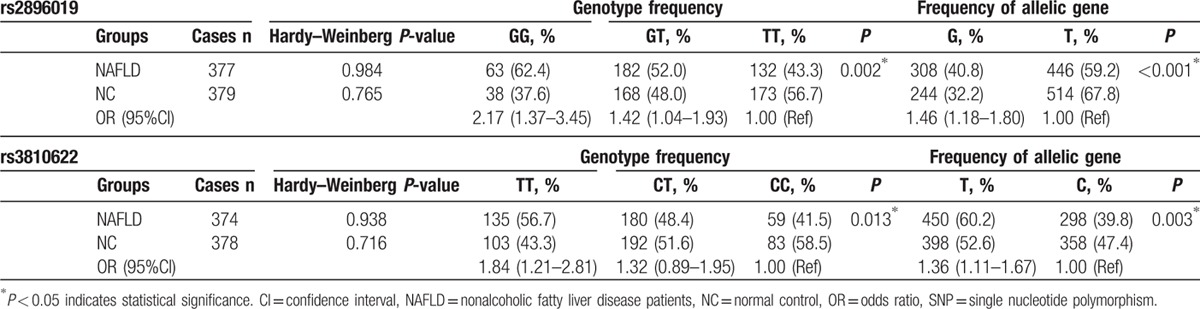
Correlation between NAFLD group and controls in genotype frequency and frequency of allelic gene at loci rs2896019 and rs3810622 of SNP.

The CC, CT, and TT genotype frequencies of the rs3810622 locus were 15.8% (59), 48.1% (180), and 36.1% (135) among the NAFLD group and 22.0% (83), 50.8% (192), and 27.2% (103) among the controls. Individuals with genotype TT of the rs3810622 had a higher incidence in NAFLD individuals than in controls (56.7% vs 48.4% and 41.5%, respectively, *P* = 0.013, Fig. 1C). Also, the prevalence of allele T was significantly higher in the NAFLD group than in the controls (60.2% vs 39.8%, *P* = 0.003, Fig. 1D).

We further analyzed the contribution of rs2896019 and rs3810622 genotype combinations using a dominant model in Table 3. Specifically, compared to the subjects with the TT homozygous alleles of rs2896019, carriers of homozygous GG and heterozygous GT genotypes had a significantly increased risk of NAFLD (adjusted OR: 1.56, 95% CI: 1.16–2.09). Compared to the subjects with the CC homozygous alleles of rs3810622, subjects with the homozygous TT and heterozygous CT genotypes had a significantly increased risk of NAFLD than subjects with the CC homozygous (adjusted OR: 1.50, 95% CI: 1.04–2.17).

**Table 3:**
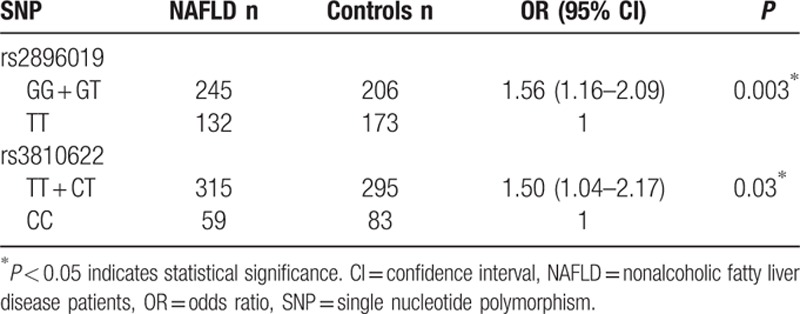
Association between rs2896019 and rs3810622 genotype combinations and NAFLD.

### 3.3 Association between genotype frequency of rs2896019 and rs3810622 and baseline clinical characteristics in NAFLD group

As shown in Table 4, we evaluated the association of rs2896019 and rs3810622 in the *PNPLA3* gene and baseline clinical characteristics in the NAFLD group. The results showed that NAFLD patients with genotype GG of rs2896019 had significantly higher LDL (*P* < 0.001), ALT (*P* = 0.003), and AST (*P* = 0.002). Moreover, NAFLD patients with genotype TT of rs3810622 had significantly higher ALT (*P* = 0.021) and GLU (*P* = 0.034), also, they had higher TG (*P* = 0.053) and LDL (*P* = 0.056), but with no statistically significance. Moreover, NAFLD patients with genotype CC of rs3810622 had significantly lower BMI than patients with genotype CT and TT (22.9 ± 23.6 vs 23.6 ± 1.6 and 23.0 ± 1.3, *P* < 0.001).

**Table 4:**
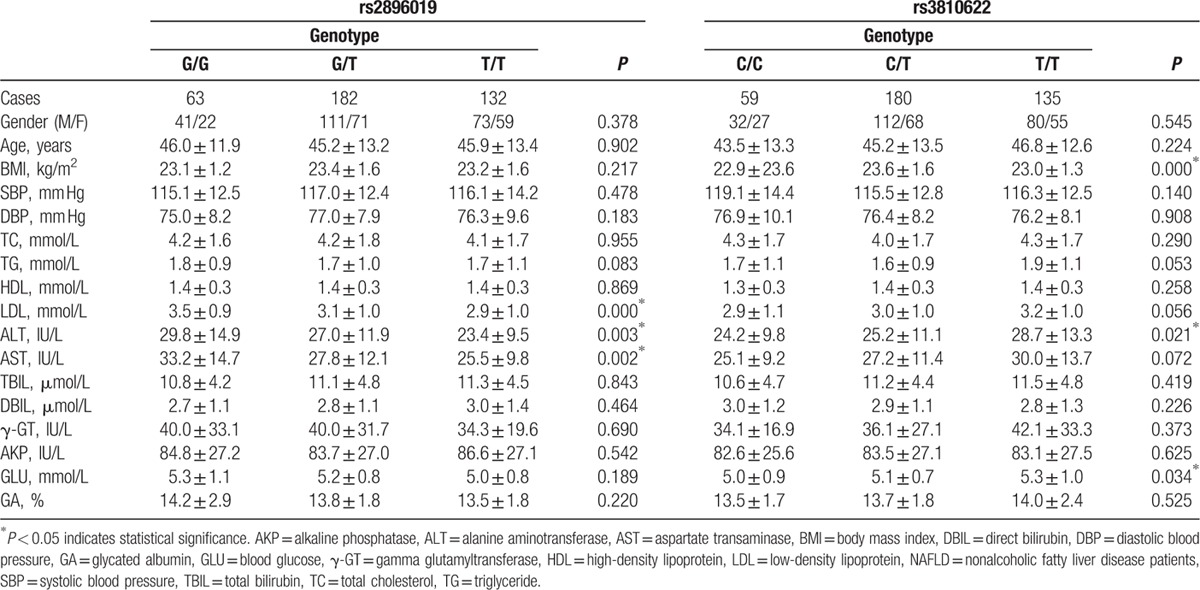
Correlation between genotypes at loci rs2896019 and rs3810622 in *PNPLA3* gene and baseline clinical information in NAFLD group.

### 3.4 Association between genotype frequency of rs2896019 and rs3810622 and fatty liver in NAFLD group

**Figure 2:**
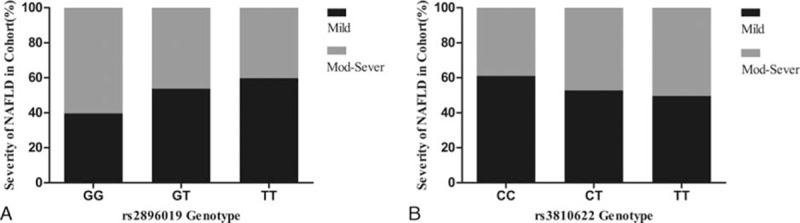
Comparison of genotype at loci rs2896019 and rs3810622 of SNP and severity of fatty liver. (A) Patients with genotype GG at locus rs2896019 had higher incidence of moderate to severe NAFLD than patients with genotype GT and TT (60.3% vs 46.2% and 40.2%, respectively, *P* = 0.03). (B) No significant association was found between genotype frequency of rs3810622 and severity of NAFLD (TT: 50.4% vs CT: 47.2% and CC: 39.0%, respectively, *P* = 0.34). NAFLD = nonalcoholic fatty liver disease patients, SNP = single nucleotide polymorphism.

The statistical analysis revealed that patients with genotype GG of rs2896019 in the *PNPLA3* gene had a higher risk of moderate to severe NAFLD than patients with genotypes GT and TT (60.3% vs 46.2% and 40.2%, respectively, *P* = 0.03, Fig. 2A, Table S2). However, no significant association was found between genotype frequency of rs3810622 and severity of NAFLD (TT: 50.4% vs CT: 47.2% and CC: 39.0%, respectively, *P* = 0.34, Fig. 2B, Table S2).

### 3.5 Haplotype analysis for rs2896019 and rs3810622

Haplotype analysis was performed to assess the effect of the combination of these 2 SNPs on NAFLD (Table 5). GT, TC, and TT haplotypes with frequency >0.03 were chosen and demonstrated that the GT haplotype was significantly correlated with a higher risk of NAFLD when compared with the healthy controls (OR = 1.49; 95% CI = 1.20–1.84, *P* < 0.01).

**Table 5:**
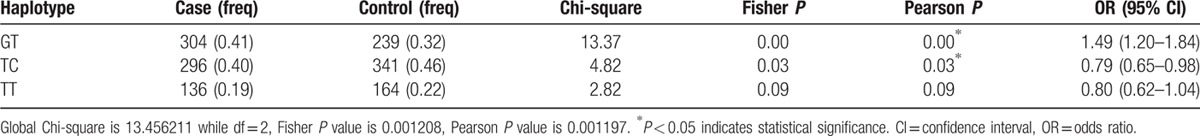
Haplotype analysis for rs2896019 and rs3810622.

## 4 Discussion

In this study, we assessed the possible association between rs2896019 and rs3810622 in *PNPLA3* gene and NAFLD in a sample of the Chinese population. The results of our study indicated that G allele of the rs2896019 locus and T allele of the rs3810622 locus were associated with the occurrence and progression of NAFLD. Furthermore, the results of haplotype analysis showed that the GT haplotype was significantly associated with the risk of NAFLD. To the best of our knowledge, this is the 1st study to assess the potential effect of rs2896019 and rs3810622 in *PNPLA3* gene on NAFLD in the Han Chinese population.

NAFLD has become one of the most common forms of chronic liver disease worldwide, and its etiology and pathogenesis are still unclear.^[17,18]^ In recent years, NAFLD is regarded as a multifactorial disease that involves a complex interaction of diet, lifestyle, and genetics.^[19,20]^ Current evidence suggests that obesity, combined with host factors such as diet, sedentary lifestyle, has been directly related to increases in the incidence of insulin resistance, which is considered as a key factor in the development of both NAFLD and NASH.^[21,22]^ Meanwhile, a healthy diet and regular exercise may enhance the insulin sensitivity and glucose homeostasis, thereby reducing the incidence of NAFLD.^[23]^ Genetic factors have also been considered to play a very important role in the NAFLD development, and multiple GWAS have discovered several genes, including TM6SF2, SAMM50, PARVB, and PNPLA3,^[13,15]^ which are potential candidates for NAFLD susceptibility and progression. Among these genes, a strong association has been demonstrated between PNPLA3 polymorphisms and NAFLD.

PNPLA3 is a membrane bound protein and considered as a triacylglycerol lipase that mediates triacylglycerol hydrolysis. Despite the physiological function of PNPLA3 has not been fully elucidated, it is generally regarded that this protein may be involved in the balance of lipid and the expression level regulated by energy intake.^[24,25]^ Also, a number of genetic variants of PNPLA3 have been found a strong connection with NAFLD, especially the rs738409 locus. Experiments with large study samples confirmed the proposed association in different ethnic populations. In recent years, other than the rs738409 locus, a variety of SNPs in PNPLA3 identified by several GWAS were revealed to be significantly associated with NAFLD. Kawaguchi et al^[8]^ conducted a GWAS study in the Japanese population and found a genetic association of rs2896019 and rs3810622 variants of the *PNPLA3* gene with pathogenic status of NAFLD. Subsequently, Kitamoto et al^[13]^ performed another GWAS that containing 3 genes (*PNPLA3*, *SAMM50*, and *PARVB*) in the Japanese population, and the results showed that rs2896019 and rs3810622 in the *PNPLA3* gene were associated with decreased serum TGs, increased AST and ALT in NAFLD patients, suggested that these 2 SNPs might play an important role in the development of NAFLD. DiStefano et al^[14]^ carried out a GWAS in obese individuals of Caucasian population, found that the rs2896019 of *PNPLA3* gene was associated with hepatic fat grades, might participate in the pathophysiology of hepatic lipid accumulation. In general, accumulating evidence has shown that the effect of PNPLA3 polymorphisms may be vital for the occurrence and development of NAFLD.

In the present study, 2 genetic polymorphisms, rs2896019 and rs3810622 in the *PNPLA3* gene, were assessed in a cohort of Han Chinese population. We found that allele G of the rs2896019 locus and allele T of the rs3810622 locus of the *PNPLA3* gene are risk alleles for NAFLD. Patients with genotype GG of the rs2896019 locus and genotype TT of the rs3810622 locus had higher incidences of NAFLD. Further investigation of the correlation between genotype frequency of these 2 SNPs and baseline clinical characteristics in NAFLD group found that patients with genotype GG of the rs2896019 locus showed more obvious lipodystrophy and liver damage. Interestingly, for these patients with TT of the rs3810622 locus exhibited higher ALT and GLU, perhaps suggesting that polymorphisms of *PNPLA3* gene may be associated with insulin resistance in addition to liver damage. Moreover, we found that patients with genotype GG of the rs2896019 locus and genotype TT of the rs3810622 locus exhibited a more severe fatty liver disease, suggesting that these 2 SNPs may probably promote the development of NAFLD. Haplotype analysis showed that GT haplotype were significantly associated with the risk of NAFLD, implying that these variants may work in conjunction in the development of NAFLD. The analysis by bioinformatics showed that these 2 SNPs were intronic polymorphisms, and they should not influence protein structure or RNA splicing. But whether transcription factors binding sites located within these 2 SNPs are still unclear, so further researches are required to clarify the mechanisms through which these 2 SNPs lead to the NAFLD.

However, several limitations of this study need to be considered. First, the diagnosis of NAFLD was primarily on the basis of ultrasonography findings. However, this is inevitable, because despite liver biopsy is the gold standard for the diagnosis of NAFLD, it is traumatic and not a routine examination for uninvestigated subjects in epidemiological studies. Second, a larger sample size from different ethnic populations is demanded to confirm our study.

In conclusion, our study shows that the rs2896019 and rs3810622 in *PNPLA3* gene contribute to increased NAFLD risk in the Han Chinese population. The present study will help determine high-risk groups and provide new ideas for the prevention and treatment of NAFLD.

## Supplementary Material

Supplemental Digital Content
