# Supplementary material for: Association of patatin-like phospholipase domain-containing protein 3 gene polymorphisms with susceptibility of nonalcoholic fatty liver disease in a Han Chinese population
Source: Medicine (Baltimore). 2016 Aug 19;95(33):e4569. doi: 10.1097/MD.0000000000004569 (PMC5370810; doi:10.1097/MD.0000000000004569)
Supplement: Supplemental Digital Content [file medi-95-e4569-s001.doc]

Table. S1 International serial number, gene name, and item number of SNPs (ABI) and the sequence of detection probe

| SNP | Gene name | Item No. | Sequence of detection probe |
| --- | --- | --- | --- |
| rs2896019 | PNPLA3 | C___1840500_10 | TGAACCTCCATCGAATGGTGCTGTA[G/T]TTTATAATGTCATCAAATATCAAAT |
| rs3810622 | PNPLA3 | C__27487068_20 | CTGCCATCTTCCCTCCTGGAGCCAC[C/T]GTGCCATGCATGGATCTGTAGCTTC |

Table. S2 Comparison of genotype at loci rs2896019 and rs3810622 of SNP and severity of fatty liver

| Loci | Genotypes | Mild fatty liver (n) | Moderate to severe  fatty liver (n) | OR(95%CI) | P |
| --- | --- | --- | --- | --- | --- |
| rs2896019 | GG (100%) | 25 (39.7%) | 38 (60.3%) | 2.27 (1.23-4.18) | 0.03 |
|  | GT (100%) | 98 (53.8%) | 84 (46.2%) | 1.28 (0.81-2.01) |  |
|  | TT (100%) | 79 (59.8%) | 53 (40.2%) | 1.00 (Ref) |  |
| rs3810622 | TT (100%) | 67 (49.6%) | 68 (50.4%) | 1.59 (0.85-2.96) | 0.34 |
|  | CT (100%) | 95 (52.8%) | 85 (47.2%) | 1.40 (0.77-2.55) |  |
|  | CC (100%) | 36 (61.0%) | 23 (39.0%) | 1.00 (Ref) |  |
